# *Glycyrrhetinic Acid*-Induced MiR-663a Alleviates Hepatic Stellate Cell Activation by Attenuating the TGF-*β*/Smad Signaling Pathway

**DOI:** 10.1155/2020/3156267

**Published:** 2020-05-11

**Authors:** Xin-Xin Guo, Wen-Na Yang, Ben-Sheng Dong, Jia-Wei Shang, Shi-Bing Su, Xiu-Li Yan, Hui Zhang

**Affiliations:** ^1^Research Center for Traditional Chinese Medicine Complexity System, Institute of Interdisciplinary Integrative Medicine Research, Shanghai University of Traditional Chinese Medicine, Shanghai 201203, China; ^2^Yueyang Hospital of Integrated Traditional Chinese and Western Medicine, Shanghai University of Traditional Chinese Medicine, Shanghai 200437, China

## Abstract

*Glycyrrhetinic acid* (GA), a hydrolysate of *glycyrrhizic acid* from licorice root extract, has been used to treat liver fibrotic diseases. However, the molecular mechanism involved in the antifibrotic effects of GA remains unclear. The involvement of miR-663a and its roles in TGF-*β*-1-induced hepatic stellate cell (HSC) activation remains unclear. In this study, we investigated the roles of miR-663a in the activation of HSCs and the antifibrosis mechanism of GA. MiR-663a expression was downregulated in TGF-*β*-treated HSCs. The overexpression of miR-663a inhibited HSC proliferation. TGF-*β*-1was confirmed as a direct target gene of miR-663a. MiR-663a alleviated HSC activation, concomitant with decreased expression of *α*-smooth muscle actin (*α*-SMA), human *α*2 (I) collagen (COL1A2), TGF-*β*1, TGF-*β*RI, Smad4, p-Smad2, and p-Smad3. GA upregulated miR-663a expression and inhibited the TGF-*β*/Smad pathway in HSCs. Further studies showed that miR-663a inhibitor treatment reversed GA-mediated downregulation of TGF-*β*1, TGF-*β*RI, Smad4, p-Smad2, p-Smad3, *α*-SMA, and CoL1A2 in TGF-*β*1-treated HSCs. These results show that miR-663a suppresses HSC proliferation and activation and the TGF-*β*/Smad signaling pathway, highlighting that miR-663a can be utilized as a therapeutic target for hepatic fibrosis. GA inhibits, at least in part, HSC proliferation and activation via targeting the miR-663a/TGF-*β*/Smad signaling pathway.

## 1. Introduction

Liver fibrosis, a major health problem worldwide, is the excessive deposition of extracellular matrix (ECM) proteins in the liver after chronic liver injury [1, 2]. Advanced liver fibrosis results in liver cirrhosis, liver failure, and portal hypertension and may need liver transplantation [3]. The activation of hepatic stellate cells (HSCs) is an important event during the progression of hepatic fibrosis. As a result of liver damage, quiescent HSCs are exposed to inflammatory cytokines and growth factors and transdifferentiate into activated myofibroblast-like cells that are characterized by the expression of alpha-smooth muscle actin (*α*-SMA) and collagen, thereby contributing to hepatic fibrogenesis [4]. Among the inflammatory cytokines and growth factors, transforming growth factor-*β*1 (TGF-*β*1) is considered to be one of the main fibrosis-stimulating factors [5]. TGF-*β*/Smad signaling plays an essential role in the development of liver fibrosis [5, 6].

MicroRNAs (miRNAs) are small (about 22 nt) RNA molecules that are associated with the posttranscriptional regulation of gene expression by affecting both the degradation and the translation of mRNAs [7, 8]. The recent findings have shown that miRNAs are important regulators of basic cellular functions, including proliferation, migration, differentiation, and apoptosis [9–11]. Some studies have demonstrated that miRNAs can modulate the activation of HSCs in hepatic fibrosis. Moreover, several studies have reported that miRNAs that modulate TGF-*β*1 expression can regulate TGF-*β*1-induced HSC activation in liver fibrosis [12–14]. For example, miR-29 regulates hepatic fibrosis through the TGF-*β*/Smad signaling pathway in HSCs [12]. MiR-146a inhibits TGF-*β*1-induced HSC activation, and miR-146a overexpression decreases TGF-*β*1-induced proliferation and increases HSC apoptosis [13]. Overexpression of miR-200a attenuates TGF-*β*1-induced HSC proliferation and activation [14]. MiR-663a is reported to induce immune dysregulation [15] and to suppress proliferation, migration, and invasion of papillary thyroid carcinoma [16], glioblastoma [17], and lung cancer [18] cells via targeting TGF-*β*1 [15–18]. Our previous study found that miR-663a is highly expressed in the serum of patients with chronic hepatitis (CHB) and miR-663a may be a potential biomarker for traditional Chinese medicine (TCM) differentiation in CHB [19]. The biological functions of miR-663a in liver diseases remain poorly understood. In our preliminary studies, miR-663a expression is downregulated in TGF-*β*1-treated HSCs, and miR-663a can inhibit HSC proliferation. Therefore, we assumed that miR-663a could target TGF-*β*/Smad signaling pathway by binding to TGF-*β*1 and inhibited HSC activation. Therefore, the role of miR-663a in fibrogenesis needs further investigation, and its link to TGF-*β*/Smad signaling also needs further assessment.

Currently, there are still no effective antifibrotic drugs approved for clinical use in humans. Therefore, it is of the utmost importance to develop effective and safe antifibrotic drugs and to reveal their underlying mechanisms. *Glycyrrhetinic acid* (GA), a hydrolysate of *glycyrrhizic acid*, is a component of licorice and is derived from licorice root extract. GA has been reported to have a wide range of pharmacological effects, including antiallergic, antiviral, and anti-inflammatory activities [20–22]. Moreover, studies indicate that GA could suppress the proliferation and activation of HSCs through the TGF-*β* signaling pathway [23, 24]. However, the molecular mechanism involved in the anti-liver fibrosis effects of GA remains unclear.

In this study, we investigated the roles of miR-663a in the activation of HSCs. MiR-663a can suppress the proliferation and activation of HSCs and the TGF-*β*/Smad pathway, highlighting that miR-663a can be used as a therapeutic target for hepatic fibrosis. We analyzed miR-663a as well as TGF-*β*/Smad signaling pathway-related protein expression in HSCs after GA treatment. We found that miR-663a was involved in the effect of GA treatment and targeted the TGF-*β*/Smad signaling pathway. Our findings suggest that the mechanism underlying GA inhibits, at least in part, HSC proliferation and activation via targeting the miR-663a/TGF-*β*/Smad signaling pathway.

## 2. Materials and Methods

### 2.1. Materials

Glycyrrhetinic acid (GA, purity 99%) was extracted and identified by the Shanghai Research Center for Modernization of Traditional Chinese Medicine (Shanghai, China). The molecular weight of GA is 470.69, and its molecular formula is C30H46O4. Antibodies against COL1A2, *α*-SMA, and TGF-*β*1 were obtained from Abcam (Cambridge, MA, USA). Antibodies targeting Smad2, Smad3, Smad4, p-Smad2, and p-Smad3 were purchased from Cell Signaling Technology (CST, Boston, MA, USA). Chemically synthesized RNAs, including miR-663a mimics, miRNA mimic negative control (miR-NC), miR-663a inhibitor (anti-miR-663a), and miRNA inhibitor negative control (anti-miR-NC), were obtained from Ambion (Thermo Fisher Scientific, USA) and Ribobio (Guangzhou RuiBio Corp., Guangzhou, China).

### 2.2. Cell Culture and Treatments

The human HSC line LX-2 was a gift from Xu et al. [25]. The human hepatocyte L02 cell line was purchased from the Cell Bank of Chinese Academy of Sciences (Shanghai, China). Cell culture conditions and cell treatments were as previously described [26]. Exponentially growing LX2 cells were treated with 10 ng/ml TGF-*β*1 (R&D Systems, Shanghai, China) for 24 h or 48 h, and then cells were collected for miRNA isolation and RT-qPCR analysis. LX2 cells were treated with GA for 48 h. The cells were then harvested for miRNA isolation for RT-qPCR analysis, and whole-cell extracts were subjected to immunoblotting analysis.

### 2.3. Transfection of miRNA Mimics and Inhibitor

The miR-663a mimics, inhibitor (anti-miR-663a), and negative controls were transfected into cells at 50 nM or 100 nM concentrations using lipofectamine 3000 (Invitrogen, Carlsbad, CA, USA) according to the manufacturer's instructions. Twenty-four hours after transfection, cells were then exposed to TGF-*β*1 (10 ng/ml) for 48 h. TGF-*β*1-treated cells were also treated with or without GA for 48 h. Cells were harvested for western blot analysis.

### 2.4. Cell Proliferation Assay

Cell proliferation was assessed with a MTS cell proliferation assay (Promega), which was conducted according to the manufacturer's instructions. For GA treatment, LX2 cells and L02 cells were plated in 96-well plates at an approximate density of 1 × 10^4^ cells/well, cultured for 24 h, and then treated with different concentrations of GA (5 *μ*M, 10 *μ*M, 15 *μ*M, 30 *μ*M, and 60 *μ*M). Cell proliferation was measured using the MTS assay at 24, 48, and 72 h.

### 2.5. RNA Isolation and Quantitative Real-Time RT-PCR Analysis

RNA isolation and quantitative real-time RT-PCR (RT-qPCR) was carried out as previously described [26]. Primers for miR-663a and U6 (RT-qPCR Primer Set) were purchased from Guangzhou RuiBio Corp. (Guangzhou, China). U6 was used to normalize the relative abundance of miR-663a. The relative mRNA expression levels were normalized to the internal control GAPDH levels. Primer sequences for target mRNAs used for RT-PCR are shown in Table 1.

### 2.6. Microarray-Based Gene Expression Analysis

Total RNA was isolated using the mirVana total RNA isolation kit (Ambion). Quantification and qualification of RNA samples were measured using a NanoDrop ND-2000 (Thermo Fisher Scientific, Inc.) and the integrity of the RNA was determined using an Agilent 2100 Bioanalyzer system (Agilent Technologies, Inc., Santa Clara, CA, USA). The microarray experiments were carried out by OeBiotech Corporation (Shanghai, China) as previously described [27]. Differentially expressed genes were selected using a fold-change (FC) in the expression of ≥2.0.

### 2.7. Western Blot Analysis

Western blot analysis was performed as previously described [25]. The relative protein expression levels were normalized to the internal control GAPDH levels. Relative protein levels were quantified using ImageJ software.

### 2.8. Bioinformatic Analysis and Luciferase Reporter Assay

To predict the potential targets of miR-663a, bioinformatics analysis was performed with TargetScanHuman 7.2 (http://www.targetscan.org/vert_72/). The procedures were performed according to the instructions on the website. According to these bioinformatics results, wild-type (WT) and mutant (MUT) seed regions of miR-663a of the full-length 3′-untranslated region (3′UTR) of TGF-*β*1 mRNA were chemically synthesized. The WT and MUT reporter gene plasmids were synthesized by Guangzhou RuiBio Corp. (Guangzhou, China). The detailed methods have previously been described [28].

### 2.9. Statistical Analysis

Measurement data were expressed as mean ± standard deviation (SD). All data were performed using GraphPad Prism 5.0 software. Statistical differences between two groups were performed by unpaired Student's *t*-test with a two-tailed distribution. Differences among multiple groups were analyzed by one-way ANOVA or two-way ANOVA. *P* < 0.05 was regarded as statistically significant.

## 3. Results

### 3.1. MiR-663a Expression Was Downregulated in TGF-*β*1-Treated HSCs, and miR-663a Inhibited HSC Proliferation

**Figure 1:**
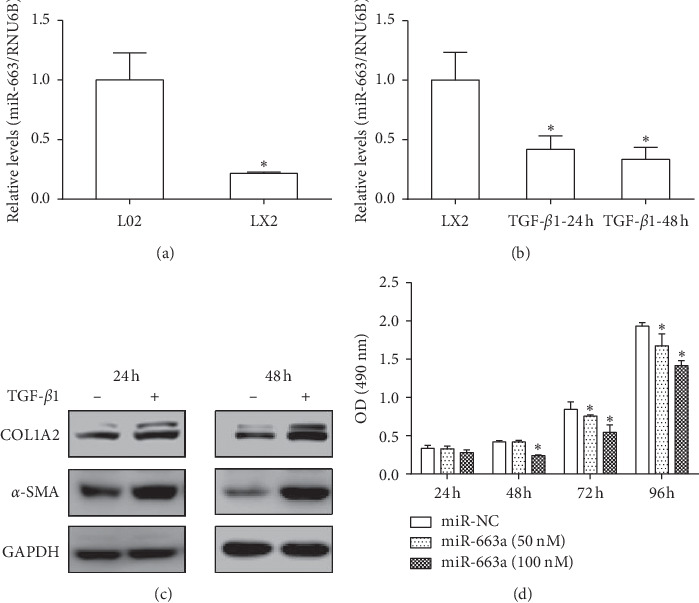
miR-663a was downregulated in TGF-*β*1-treated HSCs, and miR-663a inhibited HSC proliferation. (a) miR-663a expression was lower in LX2 cells than in L02 cells (^*∗*^*P* < 0.05). (b) and (c) MiR-663a expression (b) was inhibited, and *α*-SMA and COL1A2 protein expression levels (c) were induced by 24 h and 48 h of TGF-*β*1 (10 ng/ml) stimulation in LX2 cells. The miRNA level of miR-663a and protein levels of *α*-SMA and COL1A2 were examined by RT-qPCR and western blot. ^*∗*^*P* < 0.05, compared with LX2 cells. (d) Inhibitory effects of miR-663a overexpression on HSC proliferation. LX2 cells were transfected with miR-663a mimics or miRNA mimic negative control (miR-NC) for 24 h, 48 h, 72 h, and 96 h using lipofectamine 3000. Cell proliferation is detected by MTS. Each value is the mean ± SD of three experiments. ^*∗*^*P* < 0.05, compared with the control.

MiR-663a expression was measured in human hepatic stellate LX2 cells and normal human hepatic L02 cells. We found a significantly lower expression of miR-663a in LX2 cells than in L02 cells (Figure 1(a)). TGF-*β*1 is one of the most potent profibrogenic mediators in HSC activation, as evidenced by the stimulation of collagen production. Collagen type I alpha 2 (COL1A2) and alpha-smooth muscle actin (*α*-SMA), widely accepted as key markers of HSC activation, are often increased in activated HSCs. In this study, we assessed the stimulatory effect of TGF-*β*1 on miR-663a expression in LX2 cells. When LX2 cells were stimulated with recombinant TGF-*β*1 (10 ng/ml) at 24 h and 48 h, *α*-SMA and COL1A2 expression were significantly increased, and miR-663a expression was substantially downregulated (Figures 1(b) and 1(c)). To understand the role of miR-663a in regulating HSC proliferation, we detected the effect of miR-663a overexpression on cell growth. The HSCs that were transfected with miR-663a mimics had a significantly lower proliferation than the HSCs transfected with miR-NC at 48 h, 72 h, and 96 h (Figure 1(d)). These results indicate that miR-663a may be associated with the regulation of liver fibrogenesis.

### 3.2. TGF-*β*1 Is a Target of miR-663a in HSCs

**Figure 2:**
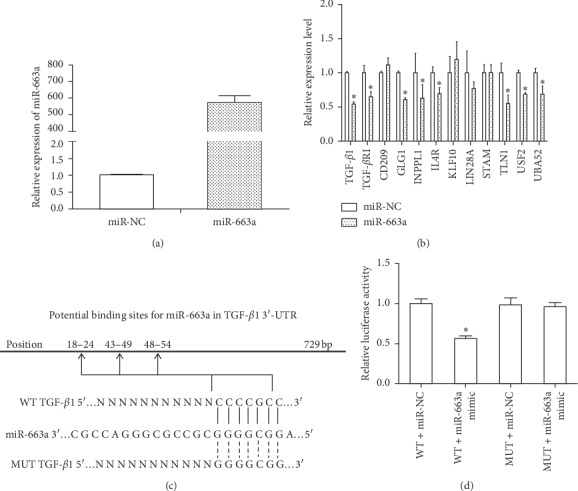
Identification of TGF-*β*1 as a direct target gene of miR-663a. (a) The relative miR-663a expression in LX2 cells was measured by RT-qPCR. LX2 cells were transfected with miR-663a mimics and miRNA mimic negative control (miR-NC) for 72 h. U6 was used as an internal control. (b) The levels of TGF-*β*1, TGF-*β*RI, CD209, GLG1, INPPL1, IL4R, KLF10, STAM, TLN1, USF2, and UBA52 were measured by RT-qPCR after LX2 cells were transfected with miR-663a mimics and miRNA mimic negative control (miR-NC) for 72 h. GAPDH was used as an internal control. The results are expressed as the mean; error bars denote the standard error of the mean. ^*∗*^*P* < 0.05, compared with the miR-NC group. (c) The three potential seed sequences of miR-663a in the 3′-UTR of TGF-*β*1 are indicated. Wild-type (WT) TGF-*β*1: the putative binding site of miR-663a on the 3′UTR of TGF-*β*1 predicted by TargetScan software. Mutant (MUT) TGF-*β*1: the mutant sequences of miR-663a seed region on the 3′-UTR of TGF-*β*1. (d) Dual-luciferase reporter assay was performed to validate the direct binding between TGF-*β*1 3′-UTR and miR-663a. After subcloning the WT or MUT of TGF-*β*1 3′-UTR into a luciferase reporter vector, we cotransfected the miR-663 mimics or miRNA mimic negative control (miR-NC) with the vector inserted with WT or MUT TGF-*β*1 3′-UTR into the LX2 cells. Then, the luciferase activity was examined. ^*∗*^*P* < 0.05, compared with the WT + miR-NC group.

To identify miR-663a-regulated genes, we generated a gene expression microarray of LX2 cells transfected with miR-663a mimic or miR-NC for 72 hours. We obtained 3610 differentially expressed genes upon miR-663a reintroduction (Figure 2(a)), with 1823 upregulated and 1787 downregulated (data not shown). We selected 10 downregulated genes for validation due to their mRNA in the 3′UTR containing the possible miR-663a binding sites by using the TargetScan database, including TGF-*β*1, CD209, GLG1, INPPL1, IL4R, KLF10, STAM, TLN1, USF2, and UBA52. Although it does not have a predicted binding site for mir-663a, we also selected TGF-*β*RI for further detection, which was also downregulated based on microarray data and was closely related to HSC activation. RT-qPCR results showed that TGF-*β*1, TGF-*β*RI, GLG1, INPPL1, IL4R, TLN1, USF2, and UBA52 were downregulated in miR-663a-overexpressing HSCs (Figure 2(b)).

Previous studies showed that TGF-*β*1 was a target of miR-663a in papillary thyroid carcinoma, glioblastoma, and lung cancer cells [15–18]. To clarify the underlying mechanisms by which miR-663a regulates HSCs senescence, we predicted the target genes of miR-663a by using a bioinformatics tool (TargetScan). Target prediction showed that the 3′-UTR of TGF-*β*1 mRNA contains putative miR-663a binding sites (Figure 2(c)). To determine whether miR-663a regulates TGF-*β*1 by binding to the corresponding 3′-UTR, we cloned the full-length 3′-UTR from TGF-*β*1 into the pmiR-RB-REPORT™ luciferase vector and cotransfected these vectors with miR-663a or control mimics into cells and evaluated TGF-*β*1 response to miR-663a by using a double luciferase reporter assay. Notably, the miR-663a mimics significantly reduced the luciferase activity driven by wild-type 3′UTR of TGF-*β*1 compared with miR-NC in HSCs but did not affect luciferase activities of mutant type TGF-*β*1 3′UTR (Figure 2(d)). Taken together, these results demonstrated that miR-663a directly targets the 3′-UTR regions of TGF-*β*1 and thus inhibits TGF-*β*1 expression at the posttranscriptional level.

### 3.3. MiR-663a Directly Obstructed the TGF-*β*/Smad Signaling Pathway in HSCs

**Figure 3:**
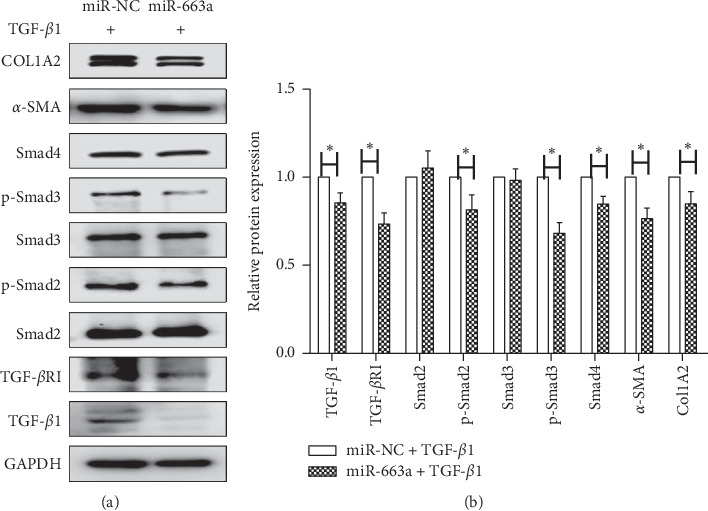
Overexpression of miR-663a inhibited the TGF-*β*/Smad signaling pathway in HSCs. (a) and (b) LX2 cells were transfected with miR-663a mimics or miRNA mimic negative control (miR-NC) for 24 h and treated with TGF-*β*1 (10 ng/ml) for 48 h. The protein expression levels of TGF-*β*1, TGF-*β*RI, Smad2, Smad3, Smad4, COL1A2, and *α*-SMA and the protein levels of p-Smad2 and p-Smad3 in the cytoplasm and nucleus were measured by western blot. Each value is the mean ± SD of three experiments. ^*∗*^*P* < 0.05, compared with the control group.

To further explore whether miR-663a is associated with the activation of HSCs, LX2 cells were transfected with miR-663a mimics and miRNA mimic negative control (miR-NC) for 24 h and then treated with TGF-*β*1 (10 ng/ml) for additional 48 h. Western blot analysis indicated that miR-663a overexpression led to the suppression of the proteins of *α*-SMA and type I collagen (Figures 3(a) and 3(b)). Our results suggest that miR-663a plays a crucial role in the suppression of HSC activation.

We next investigated the mechanisms responsible for the miR-663a-induced downregulation of *α*-SMA and COL1A2 expression. TGF-*β*1 and its downstream Smad signaling pathway represent key profibrogenic mediators. However, the involvement of miR-663a and its roles in TGF-*β*1-induced HSC activation remain unclear. We found that the overexpression of miR-663a inhibited TGF-*β*1, TGF-*β*RI, and Smad4 protein expression in TGF-*β*1-treated LX2 cells (Figures 3(a) and 3(b)). Besides, the introduction of miR-663a suppressed the phosphorylation of Smad2 and Smad3 (Figures 3(a) and 3(b)). These results demonstrate that miR-663a directly obstructs TGF-*β*1-induced HSC activation by targeting the TGF-*β*/Smad signaling pathway.

### 3.4. GA Inhibited HSC Growth and Activation

**Figure 4:**
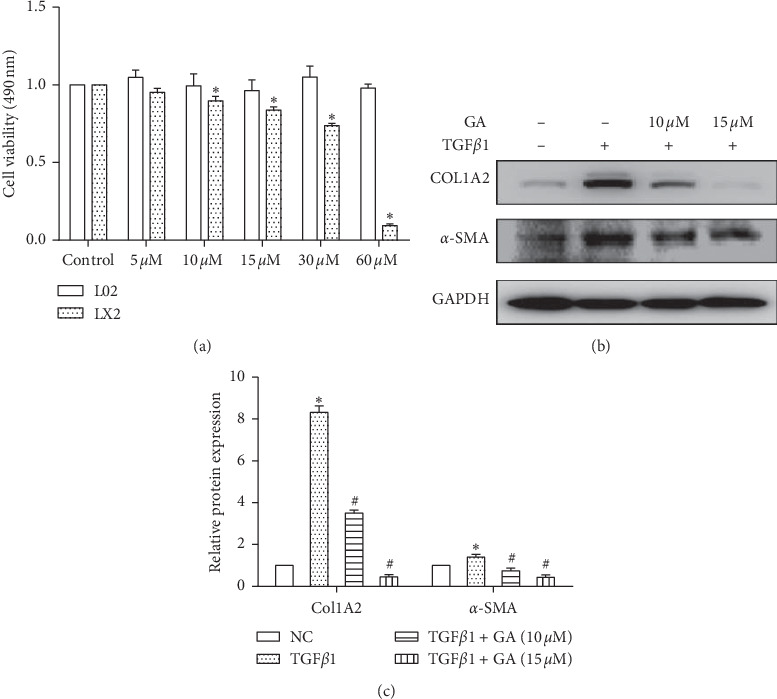
GA inhibited HSC growth and activation. (a) LX2 and L02 cell proliferation were determined by MTS assays with or without GA at the indicated concentrations (5–60 *μ*M) for 48 h ^*∗*^*P* < 0.05, compared with the untreated control cells. (b) and (c) Effect of GA on HSC activation. The protein expression of COL1A2 and *α*-SMA was significantly decreased at 48 h (*P* < 0.05) after GA treatment in LX2 cells. Each value is the mean ± SD of three experiments. ^*∗*^*P* < 0.05, compared with the untreated control cells (NC) and ^#^*P* < 0.05, compared with the TGF-*β*1 group.

To investigate the effect of GA on human HSC (LX2 cells) and human normal liver cell (L02 cells) growth, cell proliferation was measured by MTS assays in the presence or absence of GA at the indicated concentrations (5–60 *μ*M) for 48 h. As shown in Figure 4(a), at 48 h after treatment with GA at 10 *μ*M, 15 *μ*M, 30 *μ*M, and 60 *μ*M, LX2 cell growth was markedly reduced by 10.17%, 16.23%, 26.18%, and 90.61%, respectively (*P* < 0.05). However, GA did not have any effect on L02 cell proliferation. To further evaluate the effect of GA on HSC activation, serum-free medium-treated LX2 cells were cultured in DMEM or stimulated with TGF-*β*1 (10 ng/ml) in the presence or absence of GA at the indicated concentrations (10 *μ*M and 15 *μ*M) for 48 h, and COL1A2 and *α*-SMA proteins were measured. As shown in Figures 4(b) and 4(c), at 48 h after treatment with GA at 10 *μ*M and 15 *μ*M, COL1A2 and *α*-SMA protein levels were markedly reduced (*P* < 0.05). The following experiments used 15 *μ*M GA.

### 3.5. GA Upregulated miR-663a Expression and Inactivated the TGF-*β*/Smad Signaling Pathway

**Figure 5:**
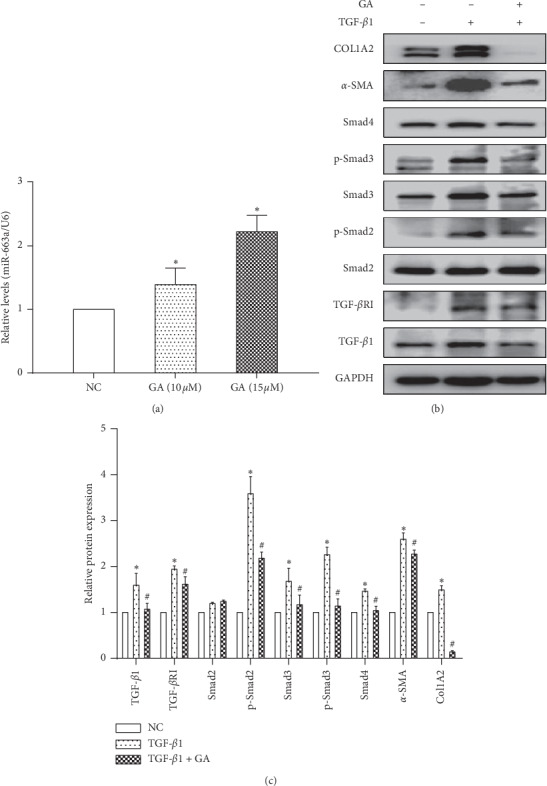
GA regulated the miR-663a/TGF-*β*/Smad signaling pathway. (a) GA significantly induced the expression of miR-663a at 10 *μ*M and 15 *μ*M in LX2 cells. MiR-663a expression was measured by RT-qPCR. U6 was used as an internal control. ^*∗*^*P* < 0.05, compared with the untreated control cells (NC). (b) and (c) The protein expression of TGF-*β*1, TGF-*β*RI, Smad3, Smad4, p-Smad2, p-Smad3, COL1A2, and *α*-SMA was markedly decreased at 48 h after GA treatment in LX2 cells. The protein expression was measured by western blot. GAPDH served as an internal control. ^*∗*^*P* < 0.05, compared with the control group, and ^#^*P* < 0.05, compared with the TGF-*β*1 group.

We next sought to examine the regulation of the miR-663a/TGF-*β*/Smad signaling pathway by GA. Undoubtedly, GA significantly induced the expression of miR-663a at 10 *μ*M and 15 *μ*M in LX2 cells (Figure 5(a)). Furthermore, GA significantly attenuated the TGF-*β*-induced elevation of *α*-SMA and COL1A2 levels (Figures 5(b) and 5(c)). To explore whether the TGF-*β*/Smad signaling pathway is involved in the effects of GA on hepatic fibrosis, the expression levels of the TGF-*β*/Smad signaling pathway-related proteins were detected in vitro after GA treatment. The protein expression of TGF-*β*1, Smad2, Smad3, Smad4, p-Smad2, and p-Smad3 was significantly increased in human HSCs stimulated with TGF-*β*1 (10 ng/ml) (Figures 5(b) and 5(c)). However, the protein expression of TGF-*β*1, TGF-*β*RI, Smad3, Smad4, p-Smad2, and p-Smad3 was significantly decreased at 48 h (*P* < 0.05) after GA (15 *μ*M) treatment in TGF-*β*1-treated LX2 cells (Figures 5(b) and 5(c)). These results showed that GA could suppress the TGF-*β*/Smad signaling pathway with an increase in the level of miR-663a.

### 3.6. GA Inhibited TGF-*β*/Smad Signaling Pathway-Related Proteins in HSCs by miR-663a Induction

**Figure 6:**
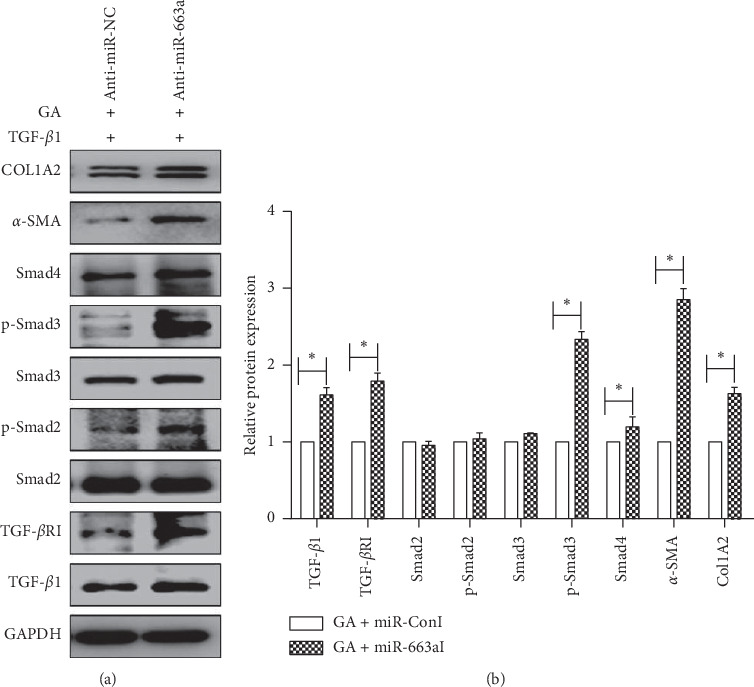
Inhibition of miR-663a overcame the GA-mediated suppression of the TGF-*β*/Smad signaling pathway. (a) and (b) The protein expression levels of TGF-*β*1, TGF-*β*RI, Smad2, p-Smad2, Smad3, p-Smad3, and Smad4 and the protein levels of COL1A2 and *α*-SMA in the cytoplasm and nucleus were detected by western blot. LX2 cells were transfected with miR-663a inhibitors (anti-miR-663a) or negative control inhibitors (anti-miR-NC) for 24 h and then treated with GA and TGF-*β*1 for 48 h. GAPDH was used as an internal control. Each value is the mean ± SD of three experiments. ^*∗*^*P* < 0.05, compared with the control group.

To further explore whether miR-663a is associated with the antifibrotic effects of GA, we transfected LX2 cells with miR-663a inhibitor and miRNA inhibitor negative control (miR-NC) for 24 h and then treated them with 15 *μ*M GA and 10 ng/ml TGF-*β*1 for 48 h. The expression of COL1A2 and *α*-SMA proteins and the expression level of TGF-*β*/Smad signaling pathway-related proteins were further identified by western blot analysis. We found that inhibition of miR-663a by miR-663a inhibitors attenuated the GA-inhibited expression of COL1A2, *α*-SMA, and TGF-*β*/Smad signaling pathway-related proteins at a 15 *μ*M dosage. Notably, the inhibition of miR-663a blocked the GA-induced downregulation of *α*-SMA and COL1A2 protein levels (Figures 6(a) and 6(b)). Moreover, decreased TGF-*β*1, TGF-*β*RI, Smad4, p-Smad2, and p-Smad3 levels in GA-treated cells were additionally reversed by miR-663a inhibitors (Figures 6(a) and 6(b)). These results indicated that miR-663a is associated with GA-inhibited TGF-*β*/Smad signaling pathway-related proteins in HSCs.

## 4. Discussion

Hepatic fibrosis, which is characterized by the excessive accumulation of dense ECM, is the primary risk factor for the development of liver cirrhosis, liver failure, and portal hypertension [1–3]. Activated HSCs have been identified as the main collagen-producing cells in the development of liver fibrosis [4]. Understanding of the molecular mechanisms of HSC activation, which plays a crucial role in liver fibrosis, remains elusive and waits to be elucidated. It contributes to a better understanding of the pathogenic mechanisms and develops more effective therapies for liver fibrosis.

Many studies have indicated that miRNAs are associated with liver pathophysiology, including HSC activation and fibrosis progression. For example, miR-29b [12], miR-146a [13], and 200a [14] may serve as a novel regulator to modulate HSC proliferation and activation by targeting the TGF-*β*1 signaling pathway. Several studies suggest that miR-663a is associated with various human diseases [15–18, 28–32]. MiR-663a has been reported to function as an oncogene that promotes the malignancy of lung cancer [18, 29], nasopharyngeal carcinoma [30], and prostate cancer [31]. MiR-663a may also act as a potential human tumor suppressor in papillary thyroid carcinoma [16], glioblastoma [17], gastric cancer [32], and colorectal carcinoma [33]. Moreover, some studies have shown that miR-663a inhibits cell migration and invasion by targeting TGF-*β*1 in papillary thyroid carcinoma, glioblastoma, and lung cancer cells [15–18]. TGF-*β*1 and TGF-*β*/Smad signaling play a crucial role in the process of hepatic fibrosis [5, 6]; however, the relationship between miR-663a and liver fibrosis remains unknown.

In this study, we found a significantly lower expression of miR-663a in LX2 cells than in L02 cells (Figure 1(a)). TGF-*β*1 stimulation significantly increased the accumulation of collagen and enhanced the expression of *α*-SMA (markers for activation of HSCs), widely accepted as key markers of HSC activation (Figure 1(c)). Notably, miR-663a expression was decreased in TGF-*β*1-activated HSCs (Figure 1(b)), and miR-663a overexpression inhibited HSC proliferation (Figure 1(d)). We explored the protective role of miR-663a in HSC and found that miR-663a overexpression could inhibit the expression levels of *α*-SMA and COL1A2 (markers for activation of HSCs) (Figures 3(a) and 3(b)), suggesting an antifibrotic role of miR-663a in HSC activation. The result suggests that miR-663a might play an essential role in liver fibrosis and that its downregulation might be associated with HSC activation.

Aberrantly activated TGF-*β*/Smad signaling is involved in the activation of HSCs and contributes to the progression of hepatic fibrosis [5, 6]. The TGF-*β* family includes multiple isoforms (TGF-*β*1, TGF-*β*2, and TGF-*β*3), and TGF-*β*1 is considered as the major profibrogenic mediator [34]. Generally, TGF-*β*1 binds to TGF-*β*RI and then recruits and activates TGF-*β*RII [34, 35]. Once TGF-*β*RI is activated, it phosphorylates Smad2/3 and then initiates TGF-*β*/Smad signaling. Inhibition of TGF-*β* or the TGF-*β*/Smad signaling shows antifibrotic effects in fibrotic diseases [35, 36]. In this study, we found that miR-663a decreased TGF-*β*1, TGF-*β*RI, Smad4, p-Smad2 and p-Smad3, *α*-SMA, and COL1A2 protein expression levels in TGF-*β*1-treated LX2 cells (Figures 3(a) and 3(b)), suggesting that miR-663a could inhibit HSC activation by targeting the TGF-*β*/Smad signaling pathway. In addition, target prediction and luciferase assays have linked the miR-663a and TGF-*β*1. Our results indicated that miR-663a could directly target TGF-*β*1 mRNA through binding to the 3′-UTR regions of TGF-*β*1, and thus TGF-*β*1 protein expression in HSCs was negatively regulated by miR-663a (Figures 2(c) and 2(d)). These results indicated a new role and mechanism of miR-663a in regulating cell proliferation during HSC activation. However, another important feature of activated HSCs is diminished cytoplasmic lipid droplets. A limitation of our study is the lack of in vivo experiments to confirm the role of miR-663a on liver steatosis and liver fibrosis. Further studies are aimed at evaluating the effect of miR-663a on lipid accumulation in HSCs, and further elucidating the underlying mechanisms.

Hepatic fibrosis is the key stage in the development of hepatic injury to cirrhosis or hepatocellular carcinoma, and there are currently no effective drugs for hepatic fibrosis. Thus, the prevention and treatment of hepatic fibrosis are a pivotal therapeutic strategy for liver disease. GA, derived from traditional medicine licorice, has been identified as a potential antihepatotoxic agent [37] and has been used in the clinical treatment of liver diseases [38]. GA can alleviate hepatocyte apoptosis through the p53 pathway to inhibit the progress of CCl4-induced hepatic fibrosis in rats [38]. GA could attenuate the mRNA and protein expression of Smad3 and types I and III collagen in human and rat HSCs, upregulate Smad7 expression, and inhibit DNA binding activities of SP-1, AP-1, and NF-*κ*B [23]. Their studies showed that GA could inhibit HSC activation and proliferation; however, the molecular mechanism for its antifibrotic effects remains unclear.

Some studies have shown that GA has protective effects in various liver damage models, such as CCl4-induced liver damage [39], free fatty acid-induced liver damage [40], and bile acid-induced liver damage [41]. In this study, we found that GA did not have any effect on the proliferation of human normal liver L02 cells (Figure 4(a)). However, GA treatment suppressed the activation of HSCs, including HSC proliferation, ECM production (COL1A2), and *α*-SMA expression (Figures 4(b) and 4(c)). To further explore whether the miR-663a/TGF-*β*/Smad pathway is associated with the effects of GA on hepatic fibrosis, miR-663a expression and the TGF-*β*/Smad signaling pathway-related protein levels were detected in vitro after GA treatment. We found that GA induced the expression of miR-663a and suppressed the TGF-*β*/Smad signaling pathway (Figures 5(a)–5(c)). The protein expression of TGF-*β*1, TGF-*β*RI, Smad3, Smad4, p-Smad2, p-Smad3, COL1A2, and *α*-SMA was significantly decreased at 48 h (0.05) after GA treatment in the TGF-*β*1-treated LX2 cells (Figures 5(b) and 5(c)). Further studies confirmed that anti-miR-663a efficiently abolished the inhibitory effect of GA on TGF-*β*1, TGF-*β*RI, Smad4, p-Smad2, p-Smad3, COL1A2, and *α*-SMA protein expression (Figures 6(a) and 6(b)).

In summary, we found that miR-663a suppresses the proliferation and activation of HSCs and the TGF-*β*/Smad pathway, highlighting that miR-663a may be used as a potential therapeutic target for hepatic fibrosis. GA inhibits, at least in part, HSC activation via targeting the miR-663a/TGF-*β*/Smad signaling pathway.

## Figures and Tables

**Table 1:** Primer sequences for RT-qPCR (human).

Name	Sequence (5′-3′)
TGF-*β*1-FP	GGCCAGATCCTGTCCAAGC
TGF-*β*1-RP	GTGGGTTTCCACCATTAGCAC
TGF-*β*1RI-FP	AAGTCATCACCTGGCCTTGG
TGF-*β*1RI-RP	GAATGACAGTGCGGTTGTGG
CD209-FP	TGCTGAGGAGCAGAACTTCC
CD209-RP	GTTGGGCTCTCCTCTGTTCC
GLG1-FP	CCAAGATGACGGCCATCATTT
GLG1-RP	AGCCGAATACTGCCACATTTC
INPPL1-FP	GTACCCTCGCTACCTCATGC
INPPL1-RP	GTCTTGGCCTTACGTGTGGA
IL4R-FP	GACGGCGAATGGAGCAGG
IL4R-RP	GGCTCCTGCAAGACCTTCAT
KLF10-FP	CACATTGCCGCACCTTTCAA
KLF10-RP	AGGATGCTGGCTGCTTTCAT
STAM-FP	GGACCCTGTAGAGTCGGTCT
STAM-RP	TCCTTAGGTCCAGTGCGAGA
TLN1-FP	TAGCCTGAAAGGGAACTCGG
TLN1-RP	CTTCCGTCCTGGGAACGTC
USF2-FP	AACACCCCGAGATGAGAGGA
USF2-RP	TGTTGATCTTGTCCCTCCGC
UBA52-FP	AAGACAAGGAGGGTATCCCAC
UBA52-RP	TGTTGTAGTCTGAGAGAGTGCG
GAPDH-FP	TGCACCACCAACTGCTTAGC
GAPDH-RP	GGCATGGACTGTGGTCATGAG

FP: forward primer; RP: reverse primer.

## Data Availability

All data included in this study are available upon request by contacting the corresponding author.
